# Association of HLA-B^∗^38:02 with Antithyroid Drug-Induced Agranulocytosis in Kinh Vietnamese Patients

**DOI:** 10.1155/2018/7965346

**Published:** 2018-07-05

**Authors:** Mai Phuong Thao, Pham Vo Anh Tuan, Le Gia Hoang Linh, Lam Van Hoang, Phan Huu Hen, Le Tuyet Hoa, Hoang Anh Vu, Do Duc Minh

**Affiliations:** ^1^Department of Physiology, Faculty of Medicine, University of Medicine and Pharmacy, Ho Chi Minh City, Vietnam; ^2^Department of Endocrinology, Cho Ray Hospital, Ho Chi Minh City, Vietnam; ^3^Center for Molecular Biomedicine, University of Medicine and Pharmacy, Ho Chi Minh City, Vietnam; ^4^Department of Internal Medicine, Pham Ngoc Thach University of Medicine, Ho Chi Minh City, Vietnam

## Abstract

**Background:**

HLA-B^∗^38:02 has been shown to be associated with antithyroid drug-induced agranulocytosis in Asian patients.

**Methods:**

HLA-B^∗^38:02 was analyzed by sequence-based typing in 21 patients who developed antithyroid drug-induced agranulocytosis and in 81 controls.

**Results:**

Frequency of HLA-B^∗^38:02 was 52.4% in agranulocytosis patients compared to 3.7% in controls (OR = 28.6, 95% CI = 6.8–120.2).

**Conclusions:**

HLA-B^∗^38:02 is a significant risk factor for agranulocytosis in Kinh Vietnamese patients treated with antithyroid drug.

## 1. Introduction

Antithyroid drugs (ATDs), including methimazole (MMI) and propylthiouracil (PTU), are the cornerstones in hyperthyroidism management worldwide. Despite their effectiveness and convenience, ATDs can cause serious adverse events such as drug-induced agranulocytosis. The risk of agranulocytosis induced by antithyroid drugs is estimated to be 0.2–0.5% and onset typically in the first 3 months of treatment [1, 2]; patients often present with symptoms of infection such as fever, chills, sore throat, and myalgias [3]. Although the incidence of agranulocytosis is rare and the reduction in granulocyte count is reversible after ATDs are discontinued, this adverse event is often accompanied by serious infection and is a life-threatening complication which has a mortality rate of up to 4-5% [4, 5]. So far, the pathogenic mechanism behind ATD-induced agranulocytosis is not well established but a genetic etiology has been indicated [4]. With the development of DNA sequencing techniques, many genetic variations have been found to be important in predicting drug adverse effects. For example, HLA-B^∗^15:02 and HLA-B^∗^58:01 have been identified as pharmacogenomic markers for serious skin reaction induced by carbamazepine and allopurinol, respectively [6, 7]. The risk factor for developing ATD-induced agranulocytosis was generally believed to occur suddenly without predictor until recently, several genome-wide association studies have described the association between HLA-B and this adverse event in Taiwanese, Hong Kong, Han Chinese, and European Caucasian [8–11]. However, in order to use this pharmacogenomic marker for clinical screening, it needs to be validated, as the allele frequency of HLA-B is different between populations. Therefore, we performed a case-control study to describe the clinical characteristics of ATD-induced agranulocytosis patients, to investigate the association between this adverse event and candidate genetic marker HLA-B^∗^38:02 and to explore the possible association between genetic carrier status and the clinical severity of ATD-induced agranulocytosis in Kinh Vietnamese population.

## 2. Materials and Methods

### 2.1. Subjects

This case-control study was conducted in 102 patients that had been treated with ATDs; among them are 21 patients who had developed ATD-induced agranulocytosis and 81 controls. All subjects were admitted to Cho Ray Hospital between October 2015 and October 2017. Patients with ATD-induced agranulocytosis were diagnosed with a granulocyte count below 0.5 × 10^9^/L after taking ATDs and recovered from agranulocytosis after the cessation of ATDs. Patients who had systemic diseases (lupus erythematosus, hepatocirrhosis) or hematological diseases (myelodysplasia, aplastic anemia) or received treatments known to affect leukocyte quantity (anticancer chemotherapy) were excluded from our study. Because agranulocytosis occurs very often in the first 12 weeks [4, 12, 13], patients are considered controls if they did not develop agranulocytosis after at least 12 weeks of ATD treatment. All of the control cases were confirmed to have normal granulocyte upon entering the study. This study was approved by the Ethics Committee of Cho Ray Hospital and Pham Ngoc Thach University of Medicine. All the subjects gave written informed consent before participating in the study. A series of clinical information including age, gender, thyroid hormone level, leukocyte count, granulocyte count, ATD type (CMZ/MMI), time to develop agranulocytosis, and clinical outcome were recorded.

### 2.2. DNA Extraction

2 milliliters of venous blood was collected from each subject using an EDTA anticoagulant tube. Genomic DNA was extracted from peripheral blood leukocytes by GeneJET Whole Blood Genomic DNA Purification Mini Kit (Thermo Fisher Scientific, Massachusetts, USA) according to the manufacturer's protocol, and samples were stored at −20°C until analyzed. The concentration of DNA solutions was adjusted to 150 ng/*μ*L and used as polymerase chain reaction (PCR) templates for HLA genotyping.

### 2.3. HLA Typing

Sequence-based typing for HLA-B was performed according to Lazaro protocol [14]. In brief, PCRs for HLA-B exon 2, 3, and the surrounding regions were performed by 5-UT-F and Bin3M13-R primers (Table 1). Amplification was carried out by Mastercycler@proS using the following conditions: one cycle at 98°C for 3 min and 40 cycles of denaturation at 98°C for 20 sec, annealing at 62°C for 20 sec, extension at 72°C for 1 min, and final extension at 72°C for 2 min. The length of amplified products was 1073 base pairs and confirmed by electrophoresis on 1% agarose gels with Diamond™ Nucleic Acid Dye (Promega, Madison, WI, USA). PCR product was cleaned with Exosap-IT glycerol solution (Thermo Fisher Scientific, Massachusetts, USA) and subsequently sequenced by BigDye® Terminator v3.1 Cycle Sequencing kit (Applied Biosystems, Foster City, CA, USA) with Seq-BIn2-R and Bin3M13-R primers (Table 1). Sequencing reactions were analyzed with ABI 3130 Genetic Analyzer (Applied Biosystems). Results were compared with the reference sequence of HLA-B^∗^38:02 from IMGT database. Only samples with 100% matching with reference sequence were considered positive. In the case of ambiguity, the results were resolved by cloning. The PCR product in the first step contained two ambiguous alleles that were separated by inserting into a pGEM-T vector-contained LacZ (pGEM®-T Easy Vector Systems, Promega); the cloning product was transformed into *E. coli* DH5*α* competent cells. The colonies were grown on LB agar plates with X-gal, IPTG, and Ampicillin, only white colonies were picked, and recombinant DNA was extracted by PureYieldTM Plasmid Miniprep System (Promega). The recombinant DNA was again performed PCR and sequenced with the same primers as in the previous step. The final sequencing data were compared with the reference sequence of HLA-B^∗^38:02.

### 2.4. Statistics

Data were analyzed using SPSS 18.0 software. The odd ratio and the numbers needed to test to prevent one case were calculated.

## 3. Results

### 3.1. Clinical Characteristics of Patients

All subjects were Kinh population in Ho Chi Minh City and the surrounding provinces in southern Vietnam. Patients overall comprised 79 females and 23 males. Among the 21 cases of agranulocytosis, 2 cases were treated with PTU and 19 were treated with MMI, reflecting that MMI is much more common than PTU as an ATD treatment in Vietnam. Basal characteristics of all subjects were shown in Table 2.

Analysis of the period from initiation of ATD therapy to the onset of agranulocytosis showed that agranulocytosis developed within 3 months in most patients (85.7%), after 3 months in two patients and after 18 months in the other. Most of the patients with agranulocytosis presented with fever (20/21) and sore throat (13/21) due to nasopharyngeal bacterial infection. Broad-spectrum antibiotic therapy is essential with cessation of the causative drug for the treatment of agranulocytosis. In most of the agranulocytosis cases, the hyperthyroidism status was not stable and FT3 and FT4 levels were significantly higher when compared to control cases.

### 3.2. Association between HLA-B^∗^38:02 and ATD-Induced Agranulocytosis

HLA-B^∗^38:02 was found in 52.4% (11/21) of patients with agranulocytosis and in 3.7% of control cases (3/81). The odds ratio for assessing the risk of HLA-related drug adverse effect was calculated (Table 3).

There are no significant differences in clinical characteristics and outcomes between HLA-B^∗^38:02 carriers and HLA-B^∗^38:02 noncarriers in agranulocytosis patients (Table 4).

Moreover, none of the subjects carry HLA-B^∗^27:05 which is a genetic predictor of ATD-induced agranulocytosis in Caucasian population. Our finding was similar to previously described HLA typing data in Kinh Vietnamese population [15].

### 3.3. Pharmacogenomic Predictor for ATD-Induced Agranulocytosis

We evaluate the prediction accuracy of HLA-B^∗^38:02 as a predictor marker for ATD-induced agranulocytosis. Assuming that the prevalence of ATD-induced agranulocytosis is 0.5% and the frequency of HLA-B^∗^38:02 in the tolerant population is 3.7% (i.e., 3/81), the sensitivity and specificity in predicting ATD-induced agranulocytosis were 52.4% (95% CI = 29.8–74.3%) and 96.3% (95% CI = 95.7–96.8%), respectively. The positive and negative predictive values were 6.6% (95% CI = 4.4%–9.9%) and 99.8% (95% CI = 99.6–99.8%). Approximately 420 cases need to be screened to prevent one case of ATD-induced agranulocytosis (i.e., 1/(0.005–0.005 × 52.4%)).

## 4. Discussion

ATDs are the common treatment modality for hyperthyroidism in Vietnam. Despite their effectiveness, ATDs are associated with a variety of minor adverse effects including urticaria, arthralgia, and gastrointestinal upset in 1% to 5% of patients as well as life-threatening complications such as agranulocytosis and hepatitis [1]. Risk factors of ATD-induced agranulocytosis have not been well established thus far because of its low incidence of occurrence.

The mechanism of agranulocytosis is still elusive, but an immune-mediated pathogenesis has been proposed [16]. The existence of complement-dependent IgM antibodies against granulocytes in the serum of a patient who was receiving ATDs and suddenly developed agranulocytosis was reported, but antibody-mediated cytotoxicity was evident only against granulocytes taken from 2 of the 8 donors, suggesting that only a subset of patients receiving ATD may be susceptible to agranulocytosis [17]. The contribution of genetics to these immunogenic abnormalities that underlie drug sensitivity has been shown in several studies in different population such as Hong Kongers, Chinese, Taiwanese, and European Caucasian. Among these population, HLA-B^∗^38:02 is consistently associated with ATD-induced agranulocytosis in Asian population while HLA-B^∗^27:05 is associated with this adverse event in Caucasian population [8–11].

Consistent with previous studies, we did not find any specific clinical factors related to ATD-induced agranulocytosis. However, there was a significant association between ATD-induced agranulocytosis and HLA-B^∗^38:02 but not HLA-B^∗^27:05. HLA-B alleles have been implicated in multiple serious drug-induced adverse events, including HLA-B^∗^57:01 in abacavir-induced hypersensitivity syndrome, HLA-B^∗^58:01 in allopurinol-induced serious cutaneous ADRs, and HLA-B^∗^15:02 in carbamazepine-induced Stevens-Johnson syndrome (SJS)/toxic epidermal necrolysis (TEN) [6, 7, 18]. Interestingly, HLA-B^∗^38 has previously been implicated in clozapine-induced agranulocytosis in Israeli Jewish schizophrenic patients [19], suggesting that HLA-B^∗^38 may be involved in agranulocytosis caused by multiple drugs.

The limitation of our study is the small number of control cases. This may not reflect the accurate frequency of HLA-B^∗^38:02 in hyperthyroidism population as the major cause of hyperthyroidism is Graves' disease, an autoimmune disease which can be related to specific HLA types. The frequency of HLA-B^∗^38:02 in the control cases, however, was similar to a normal Kinh Vietnamese population [15].

Interestingly, HLA-B^∗^38:02 seems to be associated with MMI-induced agranulocytosis only. In our study, this result was similar to the finding in Hong Kong population. However, because the number of PTU-induced agranulocytosis cases was small, further investigation is needed to confirm this finding. Besides, the association of HLA-B^∗^38:02 with antithyroid drug-induced agranulocytosis was significant but this variant only explained half of the agranulocytosis cases suggesting that other genetic markers may be associated with antithyroid drug-induced agranulocytosis in Kinh Vietnamese population. One candidate gene which may be involved in the pathophysiology of this adverse event is HLA-DRB1^∗^08:03 [8, 20]. The association of both class I and class II HLA with ATD-induced agranulocytosis advocates a complex pathological mechanism that needs to be investigated further.

## Figures and Tables

**Table 1:** Primers for HLA-B PCR and sequencing.

Primer	Sequence (5′ to 3′)
5UT-F	GACTCAGAATCTCCTCAGACGCCGA
Bin3M13-R	GGCCATCCCCGGCGACCTAT
Seq-BIn2-R	GGA TCT CGG ACC CGG AG

**Table 2:** Baseline characteristics of agranulocytosis and control cases.

Characteristic	Agranulocytosis cases *N* = 21	Control cases *N* = 81	*P* value
*Age (yr)* *Mean (SD)*	42.3 (10.1)	41 (13.8)	0.30
*Female sex, N (%)*	20 (95.2)	59 (72.8)	
*Antithyroid drug, N (%)*			0.66
PTU	2 (9.5)	8 (9.9)	
MMI	19 (90.5)	73 (90.0)	
*ATD duration, N (%)*			n/a
<3 months	18 (85.7)	0	
3–18 months	2 (9.5)	34 (42.0)	
>18 months	1 (4.8)	47 (58.0)	
*Initial ATD dose* *Mean (SD)*			
PTU (mg/d)	250 (70.7)	287.5 (115.7)	0.60
MMI (mg/d)	18.9 (5.9)	20.5 (5.7)	0.30
*Dose of ATD at the time of recruitment* *Mean (SD)*			n/a
PTU (mg/d)	250 (70.7)	75 (37.8)	
MMI (mg/d)	20.5 (5.6)	8.8 (4.8)	
*Fever, N (%)*	20 (95.2)	n/a	
*Sore throat, N (%)*	13 (62.0)	n/a	
*Mean granulocytes at the time of recruitment (cell/μL)* *Mean (SD)*	170 (35.2)	4255.3 (173.7)	n/a
*Thyroid hormone at the time of recruitment* *Median (Q1-Q3)*			
FT3 (pg/mL)	6.49 (2.7–14.45)	3.6 (2.8–7.1)	0.001
FT4 (pg/mL)	42.65 (15.07–65.83)	12.6 (8.8–25.5)	0.0001
TSH (mIU/L)	0.01 (0.004–0.14)	0.058 (0.004–2.37)	0.156
*Recover period (day)* *Median (Q1–Q3)*	5 (3–7)	n/a	
*Death, N (%)*	2 (9.5)	n/a	

ATD: antithyroid drug; PTU: propylthiouracil; MMI: methimazole.

**Table 3:** Association between HLA-B^∗^38:02 and agranulocytosis events.

HLA-B^∗^38:02	Agranulocytosis cases	Control cases	OR (95% CI)	*P* value
Positive	11	3	28.6 (6.8–120.2)	5.2 × 10^−7^
Negative	10	78		

**Table 4:** Comparison of clinical severity between HLA-B^∗^38:02 carriers and HLA-B^∗^38:02 noncarriers.

Characteristic	HLA-B^∗^38:02		*P* value
	Positive (*N* = 11)	Negative (*N* = 10)	
*Age (yr)* *Mean (SD)*	43.1 (13.1)	42.4 (8.2)	0.89
*Male to female ratio*	1 : 10	0 : 10	
*Antithyroid drug, N (%)*			
PTU (mg/d)	0 (0)	2 (20)	
MMI (mg/d)	11 (100)	8 (80)	
*Dose of ATD at the time of recruitment* *Mean (SD)*			
PTU (mg/d)	n/a	250 (70.7)	
MMI (mg/d)	21.8 (6.8)	20.0 (4.6)	
*Mean granulocytes at the time of recruitment (cell/μL)* *Mean (SD)*	162.7 (156.3)	178 (175.1)	0.83
*Thyroid hormone at the time of recruitment* *Median (Q1–Q3)*			0.2
FT3 (pg/mL)	6.7 (2.5–11.4)	5.9 (4.3–19.6)	0.5
FT4 (pg/mL)	32.6 (14.5–62.8)	37.2 (19.7–80.3)	0.6
TSH (mIU/L)	0.01 (0.004–0.01)	0.018 (0.004–0.017)	
*Recover period (day)* *Median (Q1–Q3)*	7 (4.5–7.5)	3 (3–5)	0.7
*Death, N (%)*	2 (18.2)	0 (0)	n/a

ATD: antithyroid drug; PTU: propylthiouracil; MMI: methimazole.

## Data Availability

Supporting data is available on request: please contact ducminh@ump.edu.vn.
